# Proteobacteria: A Common Factor in Human Diseases

**DOI:** 10.1155/2017/9351507

**Published:** 2017-11-02

**Authors:** G. Rizzatti, L. R. Lopetuso, G. Gibiino, C. Binda, A. Gasbarrini

**Affiliations:** Department of Internal Medicine, Gastroenterology Division, Policlinico “A. Gemelli” Hospital, Rome, Italy

## Abstract

Microbiota represents the entire microbial community present in the gut host. It serves several functions establishing a mutualistic relation with the host. Latest years have seen a burst in the number of studies focusing on this topic, in particular on intestinal diseases. In this scenario, Proteobacteria are one of the most abundant phyla, comprising several known human pathogens. This review highlights the latest findings on the role of Proteobacteria not only in intestinal but also in extraintestinal diseases. Indeed, an increasing amount of data identifies Proteobacteria as a possible microbial signature of disease. Several studies demonstrate an increased abundance of members belonging to this phylum in such conditions. Major evidences currently involve metabolic disorders and inflammatory bowel disease. However, more recent studies suggest a role also in lung diseases, such as asthma and chronic obstructive pulmonary disease, but evidences are still scant. Notably, all these conditions are sustained by various degree of inflammation, which thus represents a core aspect of Proteobacteria-related diseases.

## 1. Introduction

The gut is the most colonized human organ with up to 100 trillion microbes, about 10 times the number of the human cells [1]. At this level more than 50 phyla have been described with, however, the predominance of only 4 major phyla: Firmicutes, Bacteroidetes, Actinobacteria, and Proteobacteria [2].

Notably, gastrointestinal tract (GIT) is also colonized by fungi and virus, which constitute, respectively, the gut mycobiome and the gut virome [3–5].

Metagenomics allowed estimating the number of genes of the microbiota, the so-called microbiome, with a number exceeding by more than 150 times the human genome (about 3.3 million in comparison to about 20.000 genes in humans) [6], thus representing a real second genome for the host.

The number of microbial cells displays a positive rostrocaudal gradient along all the GIT: from about 10–10^3^ microbes per gram in stomach and duodenum, 10^4^–10^7^ microbes per gram in jejunum and ileum, to 10^11^-10^12^ microbes per gram in the colon [7, 8].

Furthermore, microbiota composition also varies in the different GI tracts: anaerobes are predominant in the colon, in particular Bacteroidetes and Lachnospiraceae families which belong to the Firmicutes phylum [9]. On the other hand, facultative anaerobes are predominant in the small intestine [9].

Microorganisms colonization of the GIT begins at birth, with a dynamic microbiota that progressively stabilizes in the first years of life [10]. In adults, microbiota reaches higher complexity increasing in diversity [11]. Finally, in the elderly, microbiota composition displays reduced diversity with predominance of Proteobacteria and a decrease in Bifidobacterium [12].

Moreover, many factors influence the microbiota composition during lifetime, the most important being diet, delivery mode, feeding type, drugs use, especially antibiotics, and, as already mentioned above, age [13, 14].

Gut microbiota performs many important functions in the host, with the establishment of a real symbiosis. These functions include metabolism and synthesis of nutrients, notably vitamin K and B group vitamins, tropism on the mucosa, drugs and toxins metabolism, and barrier functions [15]. In fact, microbiota is a component of the so-called gut barrier, a complex structure of paramount importance which serves as a frontier between the host and the environment, regulating the interaction between bacteria and host cells and modulating absorption of nutrients [16].

In this context, Proteobacteria are, as already mentioned, one of the most abundant phyla in the human gut microbiota. The name Proteobacteria was first proposed by Stackebrandt et al. in 1988 [17]. However, this grouping of bacteria was already been established by Woese in 1987 with the informal name of “purple bacteria and their relatives” [18]. The name Proteus derives from the ancient Greek god of the sea Proteus capable of assuming different shapes in regard with the high heterogeneity displayed by the bacteria belonging to this phylum [17]. A common trait of Proteobacteria is the Gram negative staining and, thus, the presence of the lipopolysaccharide in the outer membrane. Proteobacteria are currently the largest phylum within the bacteria domain. Based on phylogenetic analysis of 16S rRNA gene the Proteobacteria phylum is divided into 6 classes (previously regarded as subclasses of the phylum): Alphaproteobacteria, Betaproteobacteria, Gammaproteobacteria, Deltaproteobacteria, Epsilonproteobacteria, and Zetaproteobacteria. Considering that the classes division is based on molecular relatedness, it is not surprising that no specific morphological or physiological trait characterizes members within each class. Many common human pathogens are found in the Proteobacteria phylum: for example, the* Brucella* and* Rickettsia* genera belong to the Alphaproteobacteria class,* Bordetella* and* Neisseria* to the Betaproteobacteria class, while* Escherichia*,* Shigella*,* Salmonella*, and* Yersinia* to the Gammaproteobacteria class and, finally,* Helicobacter* to the Epsilobacteria class. In humans, Proteobacteria are present in various body sites, such as skin, oral cavity, and tongue and vaginal tract other than in the human gut and stool [19].

Aim of this paper is to review the latest findings regarding the role of Proteobacteria members in intestinal, but also extraintestinal diseases. Particular attention is given to metabolic and inflammatory disorders.

## 2. Metabolic Role

In recent years, the interest in studying the role of gut microbiota in several extraintestinal diseases has increased. Many studies found an implication of the microbiota and its alterations in many metabolic conditions, such as diabetes and glucose-intolerance, obesity, nonalcoholic steatohepatitis, and cardiovascular diseases [20–23].

Many alterations in the microbiota have been found in patients with features of the metabolic syndrome. For example, Lambeth et al. analyzed the fecal bacterial composition of patients with type 2 diabetes (T2DM), prediabetes (as defined in the American Diabetes Association position statement published in 2014 [24]), and healthy controls. They found a significant increase in an unknown genus belonging to the Enterobacteriaceae family (included in the phylum Proteobacteria), as well as an increase in the genus* Collinsella*, in the T2DM group in comparison to the other groups [25].

A connection between low-grade inflammation, sustained by lipopolysaccharides (LPS), and the development of metabolic disorders is well established [26]; however the hypothesis regarding the direct role of the microbiota in the development of this inflammatory state, called endotoxemia, is more recent and was studied, in particular, by Cani et al. [27, 28]. In fact, the authors found that the production of LPS is sustained by Gram negative bacteria in the gut and that administration of antibiotics reduced metabolic endotoxemia and cecal content of LPS [28].

Thanks to the technology innovations and the diffusion of novel techniques to analyze the microbiota, namely, 16s rRNA sequencing and metagenome sequencing, recent studies have focused on the identification of the bacteria that may be implicated in the genesis of endotoxemia and in the development of metabolic disorders. In this context, Proteobacteria were frequently found to be increased [29–31].

For example, Fei and Zhao found an increase in the Enterobacteriaceae family in an obese volunteer [32]. Moreover, after weight loss the Enterobacteriaceae population was the most affected, with a significant reduction. In addition, inoculation with a clinical isolate of the Enterobacter population in germ-free (GF) mice overcomes the resistance to the development of obesity after a high-fat diet (HFD) [32]. In fact, GM mice are usually resistant to HFD-induced obesity [33, 34].

Major insights into the role of microbiota in obesity comes from the very well-known study by Turnbaugh et al. carried in a mouse model of obesity, which demonstrated that not only did obese mice harbor a dysbiotic microbiota with an increase in Firmicutes and a reduction in Bacteroidetes, but also the obese phenotype could be transferred to germ-free mice through transplantation of fecal microbiota. In fact transfer of fecal microbiota from obese mice in germ-free mice resulted in a more pronounced increase in body weight in comparison to mice transplanted with microbiota from lean mice [35].

Further studies investigated whether the effect of microbiota transplantation could be replicated in non-germ-free mice after antibiotic treatment. The authors found similar, although less evident, changes in metabolic parameters to those achieved in germ-free mice. Accordingly, microbiota in the transplanted mice was only temporarily similar to microbiota of the donors mice, with the tendency to revert to the pretransplant status over time [36].

Finally, studies in humans provide interesting but still limited results. For example, Vrieze et al. investigated the effects of transplanting intestinal microbiota from lean donors to patients with metabolic syndrome assessing changes in the glucose metabolism [37]. Briefly, patients with metabolic syndrome were randomized to receive either microbiota from lean donors or autologous microbiota infusion; insulin sensitivity and microbiota composition were evaluated at baseline and after 6 weeks from the fecal infusion. After 6 weeks only the allogenic infusion group showed an improvement in peripheral insulin sensitivity with also a significant increase in microbiota diversity.

Moreover, various studies investigated the role of microbiota in nonalcoholic fatty liver disease (NAFLD) and nonalcoholic steatohepatitis (NASH). For example, Michail et al. examined the microbial composition of children with and without NAFLD. Authors found that patients with NAFLD had more abundant Gammaproteobacteria and Prevotella and significantly higher levels of ethanol [38]. Interestingly, previous studies found that both Gammaproteobacteria and Prevotella are associated with endogenous alcohol production [39] suggesting a mechanism for the development of liver damage.

Analysis of microbiota composition in children has demonstrated a gradual increase in Proteobacteria between healthy, obese, and NASH children [40]. When analyzing at family and genus levels, authors found that this difference was sustained by an increase in Enterobacteriaceae and* Escherichia*, respectively.

Similar results were obtained by Kapil et al. [41]. Authors investigated the role of small intestinal bacterial overgrowth (SIBO) in the development of NAFLD/NASH and they found that up to 37.5% of patients with NAFLD have SIBO. Interestingly, in accordance with the previously mentioned study,* Escherichia coli* was the most common bacterial isolate. Finally, patients with SIBO also had higher levels of endotoxemia [41].

Other authors hypothesized that gut microbiota may induce alterations in the gut-brain axis to explain its role in metabolic diseases. In fact, Vaughn et al. found that rats fed with HFD were associated not only with microbiota variations, in particular with proliferation of Proteobacteria, but also with reorganization of vagal afferents and microglia activation in the nucleus of the solitary tract, the brain center that modulates satiety [42]. Moreover, administration of antibiotic treatment was sufficient to revert the aforementioned neural reorganization in the nervous system, thus suggesting a direct role of the microbiota in this phenomenon [42].

Another important aspect is the shape of the gut microbiota by means of the diet. For example, de Filippo et al. compared the fecal microbiota of European children and that of children from Burkina Faso [43]. Authors found that microbiota of Burkina Faso children were characterized by a higher microbial richness and biodiversity and also by underrepresentation of Enterobacteriaceae in comparison to European children suggesting, again, a detrimental role of Proteobacteria and highlighting the importance of preserving microbial biodiversity [43].

Finally, Proteobacteria seems to be implicated also in cardiovascular diseases. For example, Amar et al. found that blood microbiota dysbiosis and, in particular, increase in Proteobacteria were associated with the onset of cardiovascular events in a general population after adequate adjusting for known cardiovascular risk factors, such as smoking [44]. Atherosclerotic disease is characterized by thickening of the artery intima due to accumulation of lipids and immune cells, mainly macrophages and T-cells which constitute the typical plaque. A link between atherosclerosis and infection has been postulated [45]. In particular more recent studies focused on the role of various pathogens on the components of the plaque with evidences of proatherosclerotic effects [46]. In this context, there is evidence that high levels of Proteobacteria are present within the atherosclerotic plaque [47]; thus it can be speculated that these microorganisms may have proinflammatory effects that may contribute to the activation of the plaque. Other authors, however, hypothesized that microorganisms might also contribute indirectly through mechanisms of molecular mimicry, in which case the “culprit” pathogen might not be found locally [46].

## 3. Inflammation and Inflammatory Bowel Disease

Interaction between microbiota and host cells in the gut is essential for the shaping and modulation of the immune system [48], with many studies reporting alteration in the microbiota composition in various inflammatory-sustained conditions both in animals and in humans [49]. In this context, Proteobacteria are often found to be increased in disease and have been identified by some authors as a possible marker of microbiota instability, thus, predisposing to disease onset [50].

Interestingly, a transient dominance of Proteobacteria, especially Enterobacteriaceae, was found in newborn mice, which, however, is progressively lost with age [51]. This alteration is also associated with a proinflammatory state as revealed by quantifications of common proinflammatory interleukins. Transition to a stable and less-reactive microbiota was associated with the production of specific IgA, which target especially Proteobacteria, thus suggesting an important role of B-cells in the control and modulation of the intestinal bacteria by means of immunoglobulin production. Consistently, both the absence of differentiated B-cells, as occurs in Ighm^−/−^ mice, and deficiency in IgA production lead to persistence in the Proteobacteria dominance even in adult mice with also a persistent inflammatory phenotype.

The authors, further exploring the role of B-cells, found that the production of specific IgA against Proteobacteria members is mediated by dendritic cells. Taken together, these data highlight the dramatic relevance of microbiota in the modulation of the host immune system [51].

The role of Proteobacteria in gut inflammation has been addressed in various mice models of colitis, with positive correlations [52, 53]. For example, Carvalho et al. used an inflammation-prone mouse model, namely, the Flagellin receptor TLR5-deficient mice (T5KO), to study the role of microbiota in the development of intestinal inflammation [54]. The authors found that mice progressing to colitis showed a definite microbiota signature characterized by increased levels of Proteobacteria, especially of the* Escherichia* genus [54].

Interestingly, Langgartner et al. recently found an expansion of Proteobacteria also in a mouse model for chronic psychosocial stress [55]. These data support the concept of a brain-microbiota axis, a novel concept which indicates the complex bidirectional cross-talk that occurs between these apparently segregated entities [56–58] and further implies Proteobacteria as disruptors of the intestinal homeostasis.

Inflammatory bowel diseases (IBD), mainly Crohn's disease (CD) and ulcerative colitis (UC), are chronic conditions characterized by persistent intestinal inflammation whose etiology is still unknown.

However, recent evidence indicates that they may result from an inappropriate and persistent inflammatory response to microbiota in a susceptible host [59].

Most studies focused on the variations of gut microbiota in IBD. In this context, Proteobacteria are often found to be increased in these conditions [9, 60–63], again supporting that these particular microorganisms may carry proinflammatory characteristics.

The exact mechanisms that lead to the increase in Proteobacteria during disease and, in particular, during inflammation are not completely known. However, the observation that during gut inflammation there is a decrease in obligate anaerobes and an increase in facultative anaerobes, such as Enterobacteriaceae, led to the formulation of the “oxygen hypothesis” [64]. In fact, it has been showed that under physiologic circumstances colon epithelial cells deplete oxygen levels in the lumen, at the mucosal interface, through beta-oxidation processes, thus generating an anaerobic environment [65]. On the other hand, in case of intestinal inflammation the epithelial cells reduce their capacity to undergo beta-oxidation with the consequence of an increased availability of oxygen which is thought to promote dysbiosis and it is associated with Proteobacteria bloom [66, 67]. Another important factor that might be implicated in the development of dysbiosis and in particular in Enterobacteriaceae bloom is nitrate. In fact it has been demonstrated that nitrate produced by the host during inflammatory conditions can be exploited by commensal Enterobacteriaceae which thus become predominant [68]. Further studies revealed that expression of Nos2, the gene encoding nitric oxide synthase, is inhibited by the activity of PPAR-*γ*, which, in turn, is activated by some microbiota products, such as butyrate [69]. In summary, absence of a healthy butyrate-producing microbiota leads to an increased Nos2 expression and nitrate production which finally permits the bloom of Enterobacteriaceae [69].

Furthermore, in the attempt to better identify specific Proteobacteria members associated with IBD, a new pathovar (i.e., a strain with same or similar characteristics, differentiated at infrasubspecific level from other strains of the same species or subspecies on the basis of distinctive pathogenicity) belonging to the* Escherichia* genus was identified [70]. This new pathogenic group was called adherent-invasive* E. coli* (AIEC) due to its potential to adhere and subsequently invade intestinal epithelial cells [70, 71]. Further studies revealed that AIEC were more prevalent in CD in comparison to healthy controls, were particularly associated with ileal CD, and were also found as the prevalent microorganisms colonizing ileal lesions of these patients [72].

Another study, conducted by Willing et al., also found an increased abundance of* Escherichia coli* bacteria specifically in ileal CD [73]. The “added value” of this study was the microbiota analysis of monozygotic twins in order to mitigate possible confounding factors linked to genetic variables [73]. Given these data, the authors speculate that environmental factors, specifically microbiota variations, may be more implicated in the definition of the IBD phenotype rather than genetic factors.

However, a more complex scenario is more likely, especially in the pathogenesis of IBD. For example, Knights et al. analyzing intestinal biopsies of patients with IBD demonstrated that there is a significant association between NOD2 risk allele count and relative abundance of Enterobacteriaceae [74]. These data are in line with the current concept of IBD as multifactorial disorders with a paramount role of microbiota variations in a susceptible host, as already mentioned.

## 4. Inflammation and Lung Diseases

Despite the common notion that the airway tract is a sterile environment, recent evidences demonstrate the existence of a lung microbiome, harboring around 500 species. A “core” airway microbiota was also identified in healthy lungs with the predominance of the same phyla present in the gut, in particular: Bacteroidetes, Firmicutes, and Proteobacteria [75].

Asthma and chronic obstructive pulmonary disease (COPD) are chronic inflammatory disease of the lungs. Recent studies focused on the analysis of the lung microbiome in these conditions in order to better understand the pathophysiology and, possibly, to develop new and more effective treatments. Several evidences suggest an important role of the microbiota in both diseases. For example, the use of antibiotics correlates with the risk of onset of asthma in children [76].

Moreover, comparison of bacterial composition of patients with or without asthma demonstrates, in different studies, a higher abundance of Proteobacteria in asthmatic patients [77, 78]. Other results include higher proportions of Firmicutes and Actinobacteria in healthy patients, which, however, did not reach statistical significance [78].

Smoking is the main risk factor for the development of COPD; however, not all smokers ultimately develop disease, thus suggesting that other factors might be implicated. Biedermann et al., for example, investigated the variation of microbiota composition at baseline and after smoking cessation [79]. After smoking cessation a decrease in Proteobacteria was evident. It must be stressed, however, that the analysis was carried out in stool samples, so the results involve the gut microbiota [79].

As for asthma, various studies found an increase in Proteobacteria in patients with COPD [80] and, in particular, in patients with exacerbations of disease [81]. Finally, in the same study, authors found different “microbial signatures,” or microbiome profiles, between bacterial and eosinophilic exacerbations, specifically, an increase in Proteobacteria in the former group and an increase in Firmicutes in the latter group [81].

## 5. Conclusions

Thanks to the recent advances in technology we are now able to better evaluate microbiota both in health and in disease. Research is particularly active in inflammatory disorders, such as IBD. Notably, inflammation is demonstrated to be implicated in the development of metabolic disorders, such as obesity, diabetes, and NASH/NAFLD. Many studies on these topics are based on the comparison of microbiota composition in health and disease with frequent observation of increased abundance of Proteobacteria in the latter group. Based on these data, authors have proposed that Proteobacteria may represent a “microbial signature” of disease [50]. Thus, microbiota may represent a novel target for the development of new therapeutic strategies to manage metabolic disorders. Further studies should focus on the possibility of modulating the intestinal microbiota in order to revert dysbiotic states with the ultimate aim of providing a benefit for the host. In this context, fecal microbiota transplantation has the ideal characteristics to serve this scope. The study of the lung microbiome is an expanding area of research, in the wake of the huge amount of data generated from studies of the gut microbiota. While this area is still mostly unexplored, many similarities with the gut microbiota can be found, for example, dissecting the link between inflammation and asthma or COPD.

In summary, Proteobacteria are often overrepresented in several intestinal and extraintestinal diseases, mostly with an inflammatory phenotype. While causality is yet to be proven, studies evaluating possible linking mechanisms between dysbiosis, in particular Proteobacteria, and diseases are eagerly awaited.
